# Remote sensing of environmental risk factors for malaria in different geographic contexts

**DOI:** 10.1186/s12942-021-00282-0

**Published:** 2021-06-13

**Authors:** Andrea McMahon, Abere Mihretie, Adem Agmas Ahmed, Mastewal Lake, Worku Awoke, Michael Charles Wimberly

**Affiliations:** 1 Department of Geography and Environmental Sustainability, University of Oklahoma, Norman, OK USA; 2 Health, Development, and Anti-Malaria Association, Addis Ababa, Ethiopia; 3 Malaria Control and Elimination Partnership in Africa, Bahir Dar, Ethiopia; 4 Amhara Public Health Institute, Bahir Dar, Ethiopia; 5 School of Public Health, Bahir Dar University, Bahir Dar, Ethiopia

## Abstract

**Background:**

Despite global intervention efforts, malaria remains a major public health concern in many parts of the world. Understanding geographic variation in malaria patterns and their environmental determinants can support targeting of malaria control and development of elimination strategies.

**Methods:**

We used remotely sensed environmental data to analyze the influences of environmental risk factors on malaria cases caused by *Plasmodium falciparum* and *Plasmodium vivax* from 2014 to 2017 in two geographic settings in Ethiopia. Geospatial datasets were derived from multiple sources and characterized climate, vegetation, land use, topography, and surface water. All data were summarized annually at the sub-district (*kebele*) level for each of the two study areas. We analyzed the associations between environmental data and malaria cases with Boosted Regression Tree (BRT) models.

**Results:**

We found considerable spatial variation in malaria occurrence. Spectral indices related to land cover greenness (NDVI) and moisture (NDWI) showed negative associations with malaria, as the highest malaria rates were found in landscapes with low vegetation cover and moisture during the months that follow the rainy season. Climatic factors, including precipitation and land surface temperature, had positive associations with malaria. Settlement structure also played an important role, with different effects in the two study areas. Variables related to surface water, such as irrigated agriculture, wetlands, seasonally flooded waterbodies, and height above nearest drainage did not have strong influences on malaria.

**Conclusion:**

We found different relationships between malaria and environmental conditions in two geographically distinctive areas. These results emphasize that studies of malaria-environmental relationships and predictive models of malaria occurrence should be context specific to account for such differences.

**Supplementary Information:**

The online version contains supplementary material available at 10.1186/s12942-021-00282-0.

## Introduction

According to the United Nations Sustainable Development Goals (SDGs), combatting diseases, including mosquito borne diseases such as malaria, is a high priority. In particular, malaria is the focus of ongoing efforts toward control and elimination [1–4]. There has been significant progress in reducing the burden of malaria, but it remains a major public health concern with 229 million malaria cases and 409,000 malaria deaths globally in 2019 [5]. The goal is to reduce these numbers by enabling access to prevention, diagnostic testing and treatment for all people [6]. However, global funding to achieve these goals is limited [7, 8]. It is essential to use available resources efficiently by spatially targeting prevention, control, and elimination efforts. Therefore, identifying areas with high risk of disease transmission is crucial when responding to disease outbreaks, and knowing what environmental factors drive spatial and temporal patterns of disease risk can aid in identifying disease hotspots [9–12].

Mosquito borne diseases, such as malaria, are highly sensitive to environmental conditions. Mosquito life history traits like longevity, fecundity, and biting rates are highly influenced by temperature [13] and humidity [14, 15]. The rate of pathogen development inside the mosquito (extrinsic incubation period) and transmission probabilities between human and mosquito are also influenced by temperature [13]. Larval habitats, and therefore mosquito abundance, are influenced by land cover [16, 17], hydrological setting [18] and water management for irrigation [19]. All of these factors determine the *receptivity* of an ecosystem, defined as its potential to support vector mosquitoes and malaria transmission cycles [20]. Receptivity is also affected by the *susceptibility* of humans to malaria, which depends on their exposure to mosquito bites and access to resources for malaria prevention and treatment. In addition, the *vulnerability* of a location to malaria is defined as the rate at which parasites are imported through movement of humans or mosquitoes from endemic areas [20].

The increasing availability of very-high resolution satellite data and long-term satellite records provides new opportunities for malaria research [21]. The use of geospatial environmental data to study the risk of diseases, including those transmitted by mosquitoes, has greatly expanded in recent decades [22, 23]. Satellite imagery enables the continuous monitoring of environmental conditions over large areas. The large number of available sensors allows us to measure a wide range of environmental factors that influence malaria receptivity, including meteorological factors such as temperature, humidity, and precipitation as well as landscape features related to land use, vegetation, surface water, and terrain [21]. Long-term records from moderate resolution sensors such as MODIS (1 km spatial resolution) have been used to study the influences of climate variation on seasonal and interannual variations in malaria [24, 25]. Data from high-resolution sensors such as Landsat (30 m spatial resolution) have been used to assess the effects of water, land cover, and land use on spatial patterns of mosquito vectors [26] and malaria cases [27–29]. Very-high resolution imagery from commercial satellites such as GeoEye (0.5 m spatial resolution) has been used to map individual households [30], and SPOT imagery (1.5 m spatial resolution) has been used to map larval habitats [31] in support of malaria research and control efforts. Thus, remote sensing is a useful tool for studying the effects of environmental conditions on mosquito borne diseases like malaria [24, 32–35]. In Ethiopia, such studies have established relationships between malaria risk and remotely-sensed environmental factors, such as land surface temperature [33, 36, 37], precipitation [36, 38], greenness and moisture indices [33], wetland cover [27], and distance to water bodies [36].

Identifying the environmental risk factors for malaria is challenging because malaria-environment relationships are heterogeneous across different geographic settings [39]. For example, the influence of rainfall on malaria cases in the Amazon varied between wetlands and upland regions [40], and the seasonal effects of rainfall on malaria vector larvae in Tanzania depended on the type of water body and its geomorphological setting [18]. In the Amhara region of Ethiopia, yearly fluctuations in malaria cases were influenced more by temperature in wetter climates and by precipitation and other variables sensitive to soil moisture in drier locations [41]. The accuracy of climate-driven models of malaria can be improved by identifying groups of locations that have similar climate sensitivities data and fitting a separate model for each group [24].

Malaria-environment relationships are also sensitive to spatial scale. In Ethiopia, spatial analyses of malaria often use case data that are aggregated by district (*woreda*) [42, 43], including several previous studies relating malaria cases to environmental risk factors [24, 44]. However, woredas vary considerably in size (from < 1 km^2^ in cities to > 10,000 km^2^ in some rural areas) and encompass varied climate, topography, and population density. Research conducted at the individual household level shows that overall malaria prevalence varies considerably between households [45, 46], as do the distributions of *P. falciparum* and *P. vivax* [47]. However, these household-based studies are typically limited to a few villages or to a sample of households across a larger region.

The goal of this study was to assess the potential for using satellite remote sensing data to quantify malaria receptivity across two study sites in Ethiopia. We considered a wide range of variables derived from moderate, high, and very high-resolution satellite imagery that measured climate variation, land cover and land use, surface water dynamics, and human settlements. The study used a malaria surveillance dataset that was aggregated at the scale of sub-district administrative units called *kebeles.* These are the smallest administrative units in Ethiopia and are important for the planning and implementation of malaria interventions. Previous research has shown that there is considerable kebele-level variation of malaria prevalence within the same district in the Amhara [48] and Oromia [46] regions of Ethiopia. Specific objectives were to identify the remotely sensed variables that were most effective at predicting spatial and interannual variability of malaria cases at the kebele level and to determine whether these relationships varied between two distinctive geographic settings.

## Methods

### Study area

**Fig. 1:**
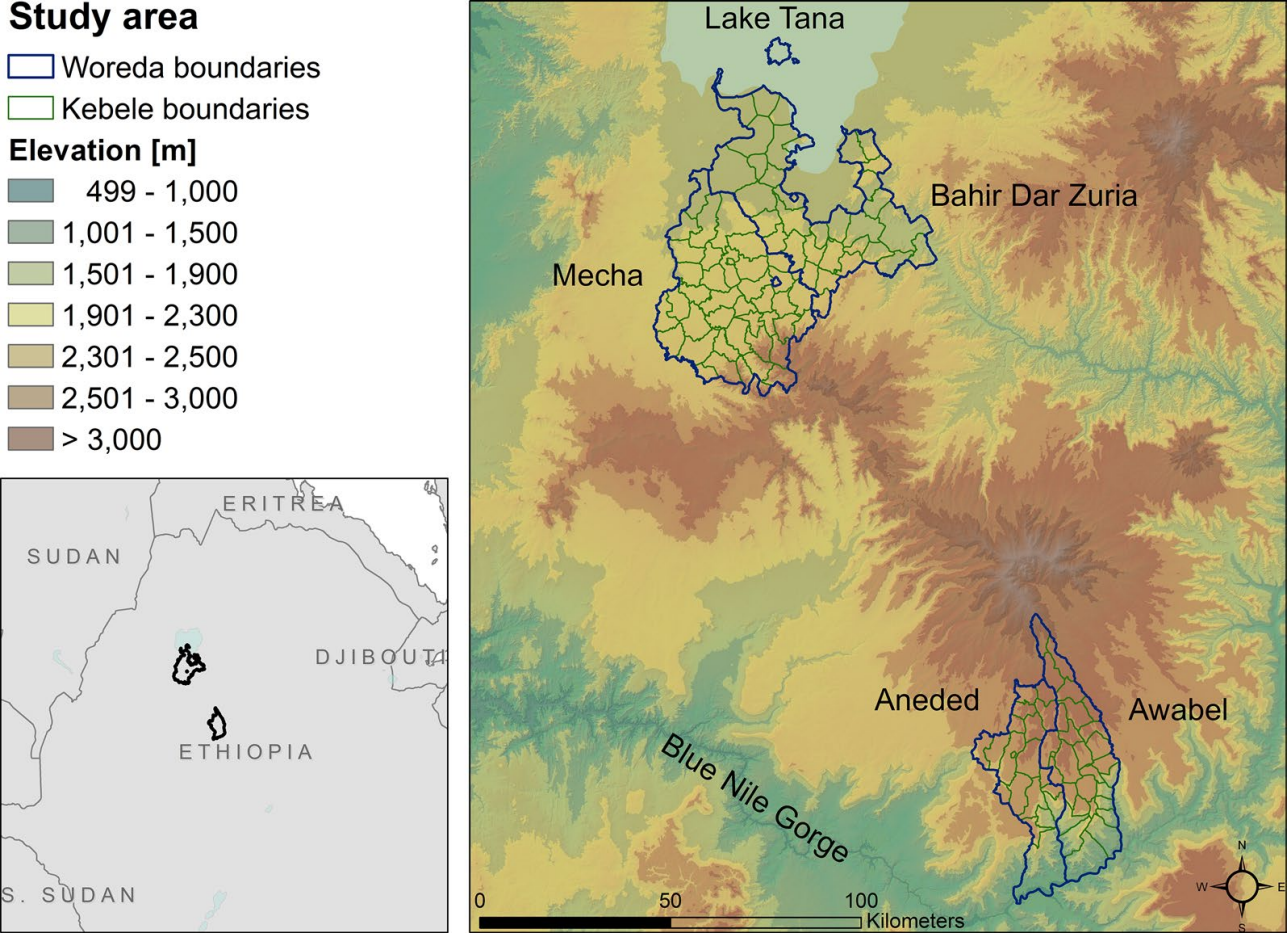
Study area in the Amhara region of Ethiopia. The left map shows the two study areas within the greater geographic area. The right map shows the two study areas and the topography of the area. Blue lines indicate woredas (district) boundaries, green lines indicate the kebeles within those woredas

Our study area covers four woredas in the Amhara region of Ethiopia that were selected because they were part of a malaria elimination demonstration project to enhance surveillance at sub-district levels (Fig. 1). The Amhara region is located in northwestern Ethiopia and is geographically very diverse. Elevation ranges from 500 to 4500 m. Climate is very seasonal with a pronounced dry season (January–April), rainy season (May–August) and a transition season (September–December). Average monthly rainfalls in Ethiopia ranges from 15 mm in January to 200 mm in July [49]. The four woredas are located in two separate areas with distinctive environmental characteristics. To explore how different environment-malaria relationships depended on geographic context, we studied each area individually.

The first study area, encompassing Mecha and Bahir Dar Zuria, is located south of Lake Tana and the city of Bahir Dar in central Amhara. Elevations range from 1500 m to 3200 m, mean daily temperatures range from 16 °C to 26 °C, and annual precipitation is between 1000 mm and 2000 mm. The northern section is relatively flat with large expanses of mixed agriculture and extensive wetlands, transitioning to the foothills of the Choke Mountains in the southern section. This study area contains a substantial area of irrigated agriculture within the Koga irrigation development project. The Koga project encompasses two dams with respective water detention ponds and water storage reservoirs. It irrigates 7004 ha of farmland via a system of canals [50].

The second study area, encompassing Aneded and Awabel, is located on the southern edge of the Amhara region between the Choke Mountains to the north and the Blue Nile River to the south. Elevations range from 1000 m to 3600 m, mean daily temperatures range from 15 °C to 28 °C, and annual precipitation is between 900 and 1300 mm. This northern section has gradually sloping terrain and is covered by a mosaic of croplands and wetlands, while the southern section drops steeply into the Blue Nile Gorge and has some croplands with large areas of bare soil and sparse vegetation. There is no large-scale irrigation scheme in this area.

The Amhara region has unstable malaria transmission that results in sporadic localized and regional outbreaks [51]. Outbreaks occur primarily between September and December. The malaria parasites in this region are *Plasmodium falciparum* and *Plasmodium vivax* [47] and the primary vector species in this region is the mosquito *Anopheles arabiensis* [52]. The Ethiopian government is committed to move towards nationwide malaria elimination by 2030 [53]. Due to successes of malaria control efforts, malaria incidence and mortality rates have been declining in the Amhara region [42] and in other parts of Ethiopia [5]. In Ethiopia, malaria surveillance data are usually reported from health facilities and then aggregated by woredas. As part of a malaria elimination pilot project, malaria data from the four woredas were tracked at the kebele level from 2013 to 2018. Kebeles are the smallest administrative unit in Ethiopia, with areas of kebeles for the entire country ranging from 0.1 km^2^ to 9500 km^2^_._ The average size is 50 km^2^ with a population of typically a few thousand inhabitants [54]. Each kebele has at least one health post that serves up to 5,000 people. Larger kebeles have health centers that serve 20,000 people. Our study area includes a total of 122 kebeles from the four study woredas: Mecha, Bahir Dar Zuria, Aneded and Awabel.

### Malaria data

Malaria case data were collected at local health posts and health centers, summarized by week for each kebele, and reported to the Amhara Regional Health Bureau. The data included malaria cases of patients who sought care at a health post or health center. As per national malaria guidelines, all suspected malaria cases at all health facilities were confirmed by multi species rapid diagnostic tests (at rural health posts) or Giemsa based microscopy (at hospitals and health centers). Weekly summaries included information on the malaria-causing pathogen (total *P. falciparum* cases, *P. vivax* cases, and mixed infections), age of the patients (above or below 5 years of age), the number of malaria patients with a travel history, and the number of total outpatients seeking care during a given week.

Because no recent population data were available at the kebele level, we calculated the proportion of outpatients diagnosed with malaria (hereafter referred to as malaria proportion). This ratio is considered a reliable indicator of malaria burden because it controls for temporal variation in health facility attendance, can be calculated in situations where accurate population data are not available [55], and has been used as a measure of malaria burden in previous studies [56–58]. Although global population datasets like WorldPop [59] and LandScan [60] are also available, we decided not to use them because we were not able to validate their accuracy and the kebele level within out study area.

The case data ranged from September 2013 to July 2018. However, we only used data from 2014 to 2017, as these were available for the entire year. Out of 472 reporting health posts or health centers, we did not have reliable location data for six. Together, these six reported only 23 malaria cases for the entire time frame and their exclusion was not expected to influence the results.

Epidemiological data were summarized for all health posts within a kebele and for each year to produce a kebele-wide annual tally of total malaria cases. For treatment and reporting purposes, malaria cases are grouped into two categories: (1) *P. falciparum* plus mixed infections, (2) *P. vivax* only. We additionally summarized total malaria cases. To detect statistically significant spatial clusters in malaria occurrence, we performed a scan statistic using SaTScan software version 9.6 [61]. We ran a retrospective purely spatial discrete Poisson model with total outpatients as the population at risk. The scan statistic was performed with an elliptical window for each year and each malaria pathogen group (total malaria cases, *P. falciparum* + mixed cases, and *P. vivax* cases) separately. After determining the spatial clusters for each year, we then identified stable hot spots (areas with recurring clusters in three or four years), unstable hot spots (clusters in one or two years), and areas that were never identified as clusters. More details on the SaTScan analysis can be found in “Additional file 1”.

### Environmental variables

**Fig. 2:**
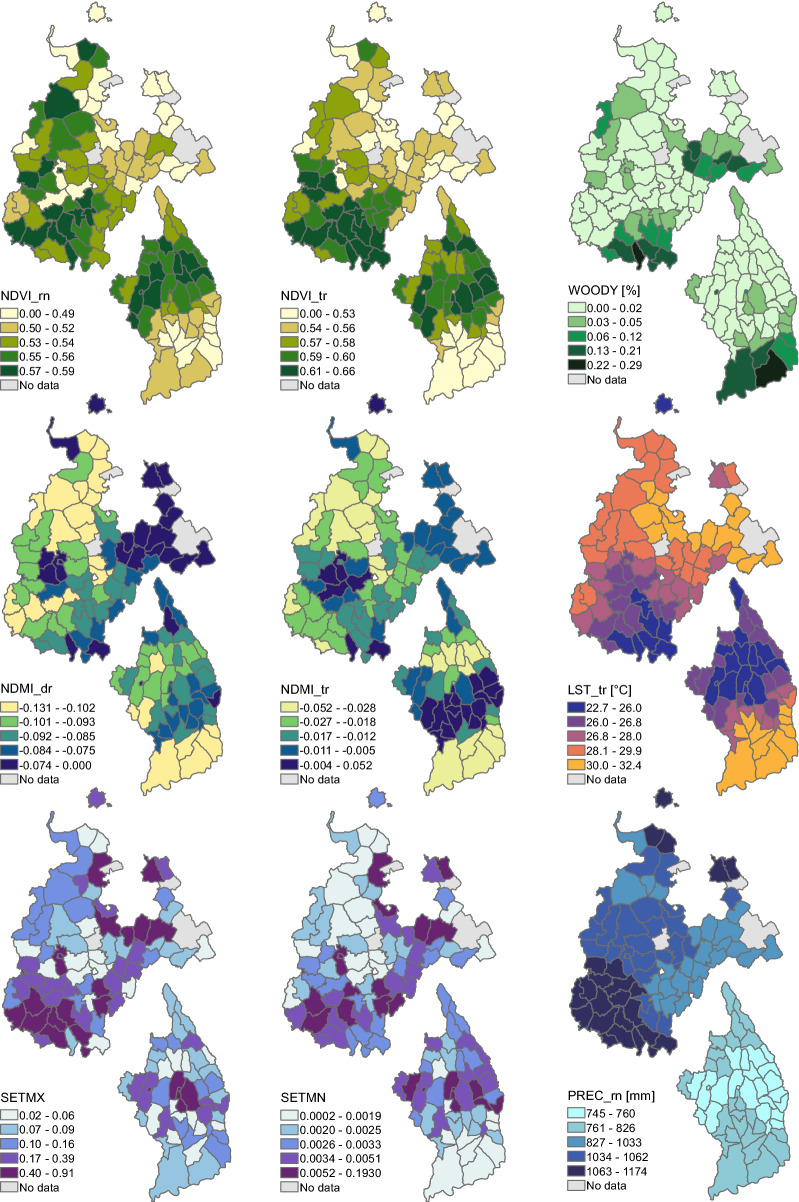
Maps displaying the variation in environmental conditions within the two study areas. Variables include NDVI for rainy season (NDVI_rn), NDVI for transition season (NDVI_tr), percent woody vegetation (WOODY), NDMI for dry season (NDMI_dr), NDMI for transition season (NDMI_tr), land surface temperature during transition season (LST_tr), maximum settlement index (SETMX), mean settlement index (SETMN), as well as precipitation during rainy season (PREC_rn). Note that the positions of the two study areas do not represent their true proximity. See the map in Fig. 1 for their actual locations.

Two types of explanatory environmental variables were used to investigate malaria case patterns: dynamic and static variables (Table 1). *Dynamic variables* included environmental conditions that were expected to vary between and within years, such as land surface temperature and remotely sensed greenness and moisture indices. *Static* variables included variables related to land cover, land use, and physiography that were not expected to vary substantially from year to year. All satellite data were reprojected to UTM zone 37 before we calculated zonal summaries for each kebele. Maps of summaries of selected variables are shown in Fig. 2. Additional baseline maps of land cover, settlements, and topography can be found in “Additional file 2”.

**Table 1:** List of static and dynamic variables used to predict malaria proportion

Type	Description	Source	Name	Units
Dynamic variables	Daytime temperature	Terra MODIS	LST	°C
	Annual rainfall	IMERG	PREC	mm
	Normalized difference vegetation index	MODIS NBAR	NDVI	Index
	Normalized difference moisture index	MODIS NBAR	NDMI	Index
	Distance to seasonal waterbodies	Landsat OLI	DISTSW	m
Static variables	Settlement mean density	PlanetScope	SETME	Index
	Settlement max density	PlanetScope	SETMX	Index
	Area below 2 m above nearest drainage	DEM, Stream network	HAND	%
	Wetland cover	Midekisa et al. [27]	WETL	%
	Woody vegetation cover	Midekisa et al. [27]	WOODY	%
	Cropland cover	Midekisa et al. [27]	CROP	%
	Open water cover	Midekisa et al. [27]	WATER	%
	Sparse vegetation cover	Midekisa et al. [27]	SPVEG	%
	Irrigation cover	Digitized from Google Earth	IRRI	%

Dynamic variables were summarized for each year for the dry season (_dr), rainy season (_rn), and transition season (_tr)

Temperature data were derived from the MODIS Terra Land Surface Temperature and Emissivity Product (MOD11A2) [62]. These data have a spatial resolution of 1 km and are provided as 8-day composites. We used daytime observations to reduce the problem of missing data from nighttime clouds. Data were filtered using the quality assurance flags to only include observations with an average LST error of below 2 °K. Temperature values were then converted to °C.

Spectral indices measuring vegetation greenness and surface moisture were derived from daily 500 m spatial resolution MODIS Nadir BRDF-Adjusted Reflectance data (MCD43A4) [63]. We applied a data quality filter using the BRDF/Albedo quality product (MCD43A2) and only included observations that were flagged as land and were “good” or “best” quality. We then calculated the Normalized Difference Vegetation Index (NDVI) [64], as well as a Normalized Difference Moisture Index (NDMI) [65].

We imputed missing values and replaced outliers from imperfect cloud-screening using a robust linear regression model from the R MASS library [66]. We fitted a robust linear regression model on our temporal data for NDVI, NDMI, and LST using cyclical splines and estimated outliers with a z-score above 3 or below − 3. We then removed observations that were identified as outliers and replaced them, as well as missing values, with predicted values from the robust linear regression model.

Precipitation data were derived from the Integrated Multi-satellitE Retrievals for Global Precipitation Measurement product (IMERG) [67]. IMERG has a spatial resolution of 0.1° (~ 11 km) and a temporal resolution of 30 min. The dynamic variables for land surface temperature, spectral indices, and precipitation were summarized for each kebele and for each season of the year: dry season (January–April), rainy season (May–August), and the transition season (September–December). For each kebele we then calculated the mean seasonal value of each dynamic variable for each year.

Because distance to potential breeding habitat has been identified as risk factor for anopheline mosquito abundance [68], we mapped seasonally flooded areas. We used 30 m Landsat 8 Operational Land Imager (OLI) imagery for the dry season and the rainy season of each year. We removed pixels that were flagged as cloud or cloud shadow and calculated the Normalized Difference Water Index (NDWI) [69] for each Landsat scene. To identify seasonally flooded areas, we extracted areas where the median NDWI during the end of the wet season (September–October) was above zero and the median NDWI during the dry season (January–April) was below zero. We then calculated the distance of each pixel to the nearest seasonally flooded pixel, expressed as cumulative cost distance of the shortest path measured in meters from the neatest water source. This was done for each year separately to account for inter-annual variation in flooding extents. Additionally, we added “year” as a continuous variable, to capture inter-annual trends of malaria proportion that are not related to environmental conditions.

S*tatic variables* measured environmental conditions that were not expected to change substantially between years. These included data on settlement structures, land cover, and the hydrological setting of the landscape. We created two settlement density indices from 3 m spatial resolution PlanetScope imagery [70]. We acquired images from November 2016 and classified each tile into building and non-building areas with a Random Forest model using a bag fraction of 0.67 over 500 classification trees. Training data consisted of sampling 7500 points collected from 2000 manually digitized polygons in each study area. The overall accuracy of the building classification based on out-of-bag data was 0.98 for both areas. In Mecha and Bahir Dar Zuria we reached a sensitivity of 0.97 and a specificity of 0.98. In Aneded and Awabel sensitivity and specificity were both 0.98. To create variables for settlement density, we resampled the classification to 100 m pixels via a majority filter and then applied a Kernel density estimator using a gaussian kernel with a radius of 100 m and a sigma of 50 m. For each kebele we summarized the mean and maximum settlement density. Settlement classification and density estimation were performed in Google Earth Engine [71].

To study the impacts of different land cover types on sub-district malaria transmission, we acquired land cover maps from Midekisa et al. [27]. This dataset provides Landsat based classifications of major land cover types in the Amhara region at a 30 m resolution. It is locally calibrated, and its overall accuracy is reported at 93.5%. We calculated the percentage of each kebele covered by the following land cover classes: open water, herbaceous wetlands, woody vegetation, cropland, and sparse vegetation.

To map areas that are likely to be flooded during larger rain events, we calculated the height above nearest drainage (HAND). The height above nearest drainage measures the vertical distance between a given point and the nearest stream. Low values indicate floodplains and other low-lying areas that are likely to be inundated during and after the rainy season when flow rates are high. We used topographic and stream network data to calculate the HAND using Topography Tools for ArcGIS [72]. We then identified areas less than 2 m above the nearest drainage and calculated the proportion of each kebele that falls into this category.

### Statistical analysis

We used Boosted Regression Trees (BRT) to model the relationships between malaria cases and environmental variables. BRT models are used in species distribution modeling [73, 74], in epidemiology [75], and to study mosquito borne diseases [32, 76–78]. Advantages of the BRT method are that it yields high accuracy while requiring little tuning [75], performs well under moderate collinearity of predictor variables [79], and captures non-linear relationships between predictor and response variables, and allows for interactions between predictor variables. Poisson BRT models were created using the R library gbm [80].

For each study area, separate models were fitted for *P. falciparum* + mixed cases, *P. vivax* cases, and total cases. Outpatient numbers were included as offset term. The models were fitted with a learning rate of 0.01, a tree complexity of 3, and a bag fraction of 0.5. Several parameter combinations were tested and the combination that yielded the best R^2^ from a fivefold cross validation was selected.

Relative importance of predictor variables was estimated to determine which variables had the strongest influences on malaria patterns. Variable importance is a measure of how often a variable is used to create a split, normalized by the improvement in squared error resulting from the corresponding split. A high variable importance of a variable indicates that the BRT model relied heavily on a specific variable during the model fitting process. It is a common metric to compare the relative influence of predictor variables [74], and has been used to study environmental effects on malaria transmission [32]. We then ranked the variables by their importance value and identified the five most important variables for total malaria cases. To visualize the relationships between these variables and malaria proportion, we fitted partial dependence plots that show how a response variable depends on the predictor variable after taking the average effects of all other variables into account.

## Results

### Spatio-temporal patterns in malaria cases

Between January 2014 and December 2017, a total of 22,584 malaria cases were reported (Table 2). Fifty-nine percent of all cases were attributed to *P. falciparum* + mixed, and 41% of cases were due to *P. vivax*. In Bahir Dar Zuria and Mecha*, P. falciparum* + mixed cases made up the largest share of infections, whereas *P. vivax* was more dominant in Aneded and Awabel. Of the four woredas, the highest malaria proportion was in Awabel with 62 malaria cases per 1000 outpatients. A total of 2108 cases had a travel history, meaning they left the village for at least one night within the last 30 days. The highest proportion of traveled cases was in Awabel with 6 traveled cases per 1000 outpatients.

**Table 2:** Total malaria case numbers, as well as case number by species, from 2014 to 2017

Woredas	Total malaria cases	*P. falciparum* + mixed cases		*P. vivax* cases		Traveled cases		Outpatients
	Count	Count	% of total cases	Count	% of total cases	Count	% of total cases	Count
Aneded	3993	2069	51.8	1924	48.2	201	5.0	79,394
Awabel	6110	3436	56.2	2674	43.8	592	9.6	97,052
Bahir Dar Zuria	6145	3767	61.3	2378	38.7	257	4.1	324,769
Mecha	6336	3975	62.7	2361	37.3	1058	16.7	416,009

The number of traveled cases represents all diagnosed malaria cases that indicated a travel history. Outpatient numbers were the total numbers of patients seeking care at health facilities. Outpatient numbers were used to calculate the proportion of outpatients diagnosed with malaria (the malaria proportion)

**Fig. 3:**
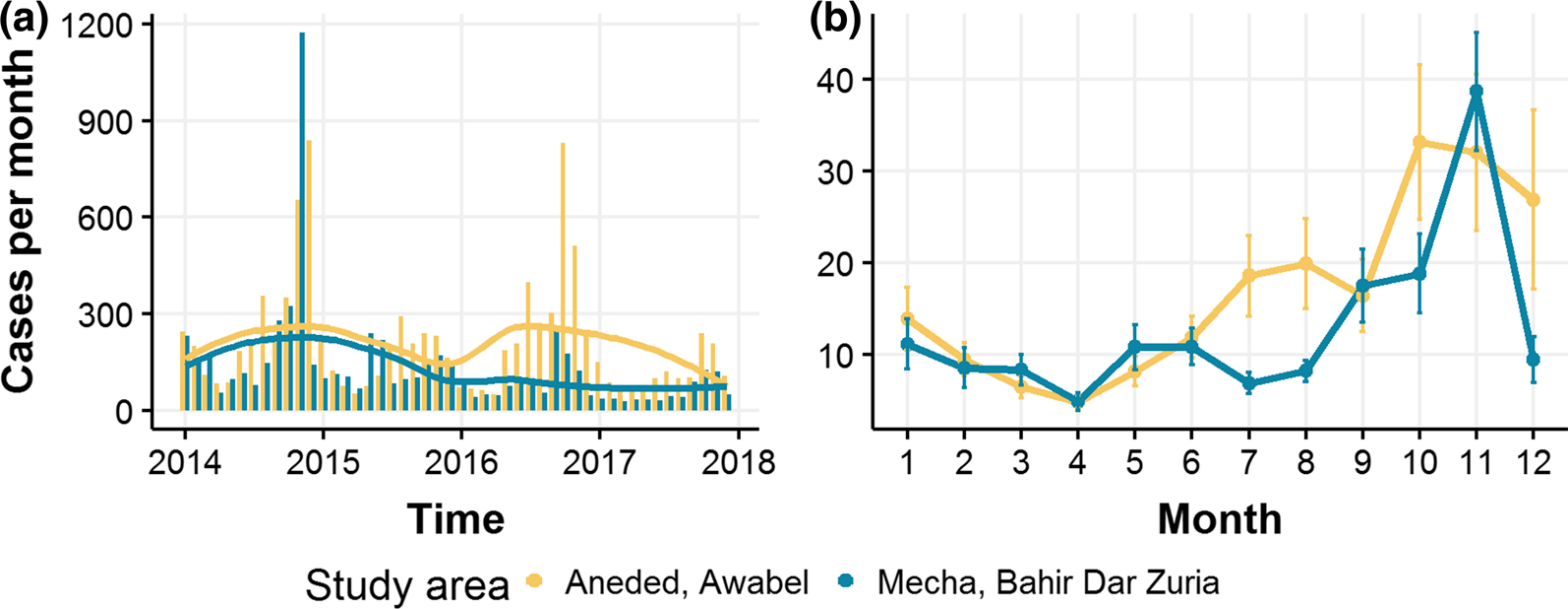
Total malaria case numbers from Jan 2014–Dec 2017 by study area. **a** Monthly case numbers shown over entire study period. Lines indicate LOESS smoothed trend. **b** Seasonality of total malaria cases. Lines represent mean case numbers over all kebeles and years. Error bars indicate the standard error of the observed mean

From 2014 to 2017, we identified a clear downward trend in total malaria cases for Mecha and Bahir Dar Zuria (Fig. 3). Case numbers in Aneded and Awabel remained more stable over the study period. This led to case numbers in Mecha and Bahir Dar Zuria being higher than those in Aneded and Awabel during 2014 and 2015, but lower in 2016 and 2017. Both study areas showed strong seasonality with low case occurrence between January and April, a small case peak during May–August and most cases occurring during September–December.

**Fig. 4:**
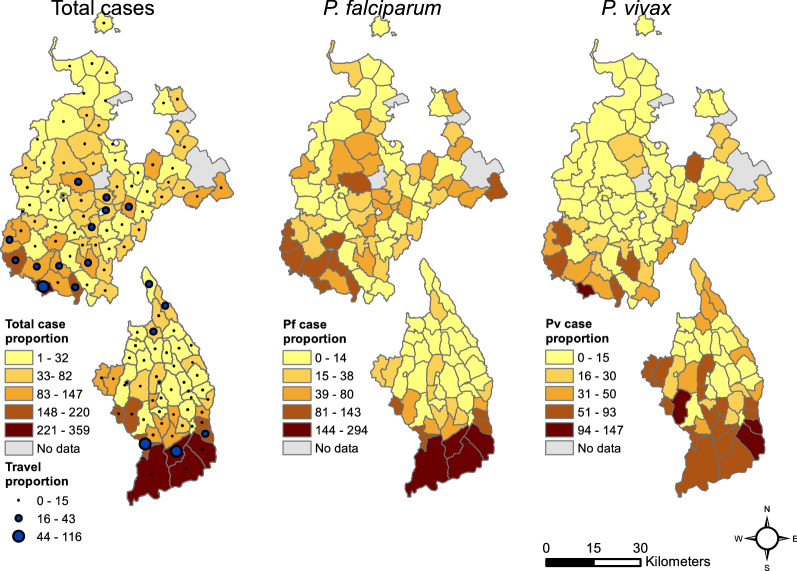
Spatial distribution of malaria proportion. Malaria proportion is the number out of 1,000 outpatients that were diagnosed with malaria for total malaria cases, *P. falciparum* + mixed cases, and *P. vivax* cases. The upper maps are the woredas Mecha and Bahir Dar Zuria, and the lower maps are the woredas Aneded and Awabel. Blue dots indicate the travel proportion, which is the total malaria patients with travel history per 1000 outpatients

There was considerable variation in total malaria proportion within woredas, as well as for cases broken up into the two pathogen groups *P. falciparum* + mixed and *P. vivax* (Fig. 4). In Mecha and Bahir Dar Zuria, there was a high malaria proportion in the hilly southern part of the study area, as well as in the flatter central area. In Aneded and Awabel there was a much stronger spatial gradient in malaria proportion with southern kebeles on the Blue Nile escarpment showing a higher malaria proportion. In Mecha and Bahir Dar Zuria, *P. vivax* was more confined to the kebeles in the southern hills, whereas *P. falciparum* cases were largely responsible for the central cluster. In Aneded and Awabel, *P. falciparum* malaria proportion was highest in the kebeles along the Blue Nile escarpment in the south, whereas infections from *P. vivax* cases had additional clusters in the northern and western kebeles. Most cases with a travel history were recorded in the southern hills of Mecha and on the Blue Nile escarpment in Aneded and Awabel. High malaria proportion was correlated with a high proportion of cases with a travel history. The Spearman rank correlation coefficient between travel proportion and *P. falciparum* proportion was 0.6 for Mecha and Bahir Dar Zuria, and 0.62 for Aneded and Awabel. The correlation between travel proportion and *P. vivax* proportion was 0.58 for Mecha and Bahir Dar and 0.54 for Aneded and Awabel.

**Fig. 5:**
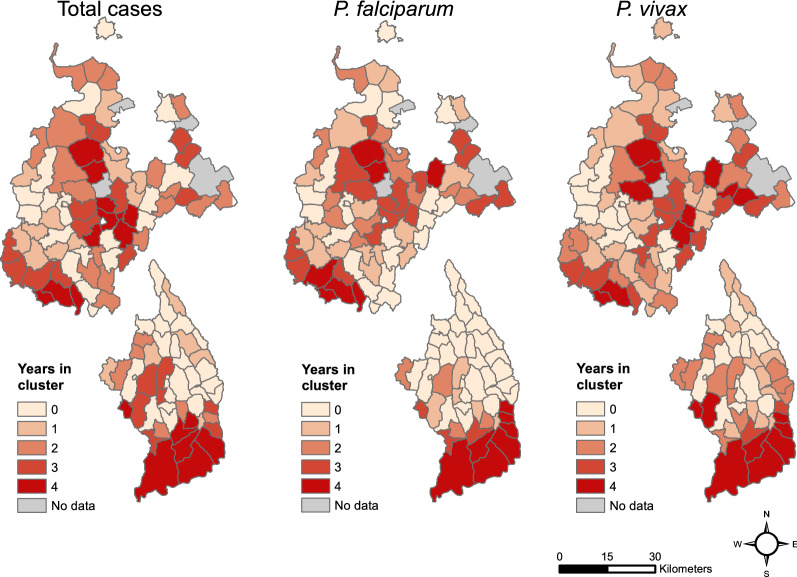
Number of years each kebele was identified as part of a case cluster by the SaTScan analyses

These general patterns were consistent with the results from the SaTScan analysis (Fig. 5). The kebeles in southern and central Mecha and Bahir Dar Zuria that showed a high malaria proportion over the entire time period were identified as stable malaria hotspots with malaria clusters in at least 3 years. Similarly, in Aneded and Awabel, areas with overall high malaria proportion were found to be stable malaria hotspots with clusters identified for every year. The SaTScan results showed that the high malaria proportion in the southern kebeles of both zones were due to consistent annually recurring outbreaks and not due to individual large outbreaks. Notably, in Aneded and Awabel most kebeles were either identified as stable hotspots for *P. falciparum* with clusters in three or four years, or as areas that were never identified as hotspots. Only very few kebeles were identified as unstable *P. falciparum* hotspots with clusters in one or two years. *P. vivax* case clusters in Aneded and Awabel were less stable with a considerable number of kebeles being identified as clusters in one or two years.

### Association between malaria and environmental variables

We compared model performance metrics for BRT models for each study area and malaria species, as well as total cases. The cross-validated R^2^ values were higher in Aneded and Awabel with 0.90 for total cases, 0.91 for *P. falciparum* and mixed cases, and 0.78 for *P. vivax* cases. In Mecha and Bahir Dar Zuria, the cross-validated R^2^ values were 0.6 for total cases, 0.54 for *P. falciparum* and mixed cases and 0.68 for *P. vivax* cases. In Mecha and Bahir Dar Zuria, the cross-validated R^2^ for *P. vivax* was higher than for *P. falciparum*, whereas in Aneded and Awabel the cross-validated R^2^ for *P. falciparum* was higher than for *P. vivax*.

**Fig. 6:**
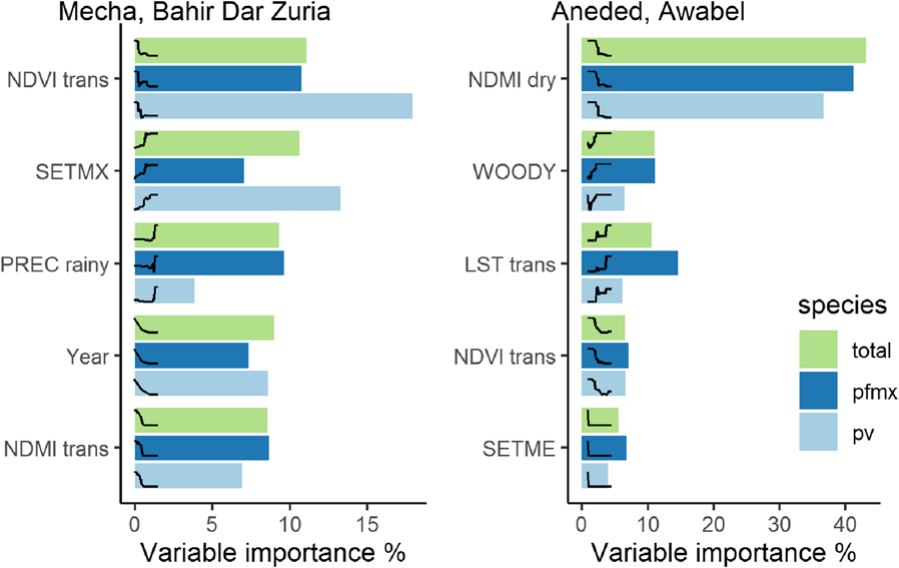
Variable importance of the five most important environmental variables as identified by boosted regression tree models. Different colored bars represent different malaria species (green = total species, dark blue = *P. falciparum* and mixed infections, light blue = *P. vivax*). Curves represent the fitted partial dependence curve for each variable

We quantified the relative importance of all variables in the different BRT models and visualized a subset of variables that represent the five most important variables for total malaria cases, as well as the respective partial dependence plots to interpret the influence of environmental variables on malaria proportion (Fig. 6). Tables with all variables and their relative importance scores can be found in the supplement “Additional file 3”. The most important variables differed between the two study areas with only NDVI during the transition season appearing in the top five of both study areas and both malaria species. In both study areas at least one of the two seasonal vegetation or moisture indices was important, as well as was at least one settlement density index. Except for % Woody vegetation, none of the land cover variables contributed substantially to any of the models. None of the hydrological variables, including the distance to seasonal water, height above nearest drainage, or percent of land within the Koga irrigation scheme, were among the most important variables.

In Mecha and Bahir Dar Zuria, all variable importance values were below 20%, indicating that the BRT model relied relatively evenly on a larger number of variables and that no variable stood out in explaining most of the variation on its own. *P. falciparum* was most strongly influenced by NDVI during the transition season (10%), with higher malaria proportion coinciding with NDVI values below 0.5. Similarly, NDMI values below -0.01 during the transition season were also associated with a higher malaria proportion. Precipitation above 1,200 mm during the rainy season was associated with a higher malaria proportion. *P. vivax* in Mecha and Bahir Dar Zuria was mostly influenced by NDVI during transition season (17%) with a higher malaria proportion related to lower NDVI values, and maximum settlement index (13%) with a higher malaria proportion in kebeles with the highest aggregations of buildings. Year was an important variable for both malaria pathogens in Mecha and Bahir Dar Zuria, indicating that the downward trend over the entire study period was driven by processes not related to environmental factors included in this model.

In Aneded and Awabel, NDMI during the dry season was by far the most important variable for both malaria species (41% for *P. falciparum* and 36% for *P. vivax*). Additionally, NDVI values below 0.55 during transition season were also associated with more malaria for both species (both < 10%). Both spectral indices showed associations of drier or less green conditions with malaria occurrence. Higher malaria proportion was also positively associated with percent cover of woody vegetation for *P. falciparum* (11%), and *P. vivax* (6%). Mean LST above 25 °C during transition season had a positive effect on malaria proportion for both species. However, the relative importance of LST for *P. falciparum* and mixed cases (14%) was larger than for *P. vivax* (6%). For both species, a mean settlement index of close to zero was associated with a higher malaria proportion (both < 10%).

## Discussion

This study presents a kebele-level analysis of malaria occurrence in the Amhara region in Ethiopia between 2014 and 2017. Remotely sensed environmental variables were used to explore the influences of environmental risk factors. We found considerable small-scale variation in the proportion of outpatients with malaria and malaria case clusters among the kebeles of each study area. Climatic variables, settlement structure, and spectral indices were associated with malaria risk, whereas variables related to seasonal waterbodies, flood plains, and irrigation had weaker relationships. However, the relative importance of the variables, as well as the association between those variables and malaria, varied between the two study areas, as well as between the different *Plasmodium* species. These results support the findings of previous studies that there is geographic variation in the relationships between environmental conditions and malaria.[18, 24, 40, 41] and highlights the importance of stratifying disease risk models into zones with similar geographic context when studying large, heterogeneous areas [24].

In Mecha and Bahir Dar Zuria, the malaria cases varied along a precipitation gradient, with the highest malaria risk in areas with the most rainfall. This effect is not surprising, as the association of malaria with climatic factors, including rainfall, has been well established in the area [33, 36, 38]. The negative associations with NDVI and NDMI values during transition season could be an indicator of available breeding sites for *An. arabiensis*. Areas that received heavy rainfall during the rainy season, but had drier conditions during the transition season, could have more isolated pools as surface water recedes. Such areas have been found to be favorable breeding sites for *An. arabiensis* [81]. Additionally, a high vegetation cover can create unfavorable conditions for *An. arabiensis*, which prefers sun-lit pools with low vegetation cover [82]. The positive influence of maximum settlement index indicates that kebeles where buildings are aggregated into one or more settlements are at higher risk than kebeles where dwellings are dispersed throughout the countryside.

In Aneded and Awabel, the malaria proportion was primarily associated with low NDMI values during the dry season. These dry areas are also located on the Blue Nile escarpment, which is the most isolated and sparsely settled part of this study area. In addition to the mosquito habitat associations discussed for Mecha and Bahir Dar Zuria, the negative influences of NDMI on malaria in Aneded and Awabel could also reflect underlying socio-economic factors. For example, there is a relationship between the risk of getting infected with malaria and the main source of water of a household, with households that need less time to access water being at lower risk than households that require more time to access water [83]. This effect may be amplified in drier environments with less water availability. Additionally, the driest kebeles in this region are sparsely settled, as reflected in the negative association between mean settlement index and malaria. Further, the terrain of the escarpment is steep and rugged and is isolated from major highways and cities. Households on the escarpment may have difficulties in accessing resources known to reduce infection risk, such as information about malaria treatment [84], bed nets [85], and other malaria prevention tools [86], resulting in higher susceptibility to malaria infections.

Land surface temperature was found to be the second strongest predictor of *P. falciparum* in Aneded and Awabel. However, it was less important in predicting *P. vivax* distribution. Previous research has found that due to the different biology of the two parasites, *P. falciparum* is limited to lower elevations with warmer temperatures, whereas *P. vivax* is more tolerant of lower temperatures and therefore occurs in higher elevations in the Amhara region [47, 87]. The effects of temperature on *P. falciparum* in Aneded and Awabel may therefore be due to a temperature threshold that is reached in the higher elevations of this study area. Additionally, in other parts of Ethiopia it has been reported that *An. arabiensis* is mostly prevalent in lower elevations whereas *An. cinereus* dominates at higher elevations [88]. Because the temperature gradient in Aneded and Awabel follows an elevation gradient, change in the dominant vector species may be an additional explanation of why we see a temperature effect in this region.

In both study areas we found that malaria cases with recorded travel histories were concentrated in certain kebeles, and these kebeles tended to have high *P. falciparum* cases. Travel has been shown to be a significant risk factor for malaria transmission, particularly for *P. falciparum* related infections [89]. In rural areas in Amhara seasonal migrants often leave their village to seek an additional income elsewhere [90]. Migrant workers are often found to have increased exposure to malaria risk due to low bed net utilization, as well as sleeping outside [91]. Migrant workers from rural areas could therefore be a source of repeated pathogen introduction into their home communities. From the available data we could not tell where and for how long they traveled, nor could we derive which *Plasmodium* species the patients with travel history were infected with. Travel history was therefore not included in our formal analysis. However, these data suggest that vulnerability to imported cases is an important factor that must be considered in addition to local receptivity for malaria transmission. Some of the observed relationships with remotely sensed environmental variables may reflect environmental settings with marginal agriculture where residents are more likely to travel for seasonal employment.

Surface water-related variables did not strongly influence malaria proportion in our model in either of the two study areas. These included the coverage of open water, herbaceous wetlands, percent of land within the Koga irrigation scheme, the average distance to the nearest seasonal waterbody, as well as areas likely to flood due to their height above the nearest drainage. These findings contrast with the general expectation of relationships between malaria cases and temporary water bodies. Other studies from different parts of Ethiopia have found that woredas with high wetland cover had high malaria incidence [27] and household surveys showed that the distance to breeding sites for samples of individual households influences malaria risk [38, 92, 93]. However, those previous studies have been performed at different spatial scales, and water-related variables were much less important at the kebele level within the woredas that we studied. Additionally, the water variables were derived from 30 m Landsat imagery and a 30 m digital terrain model, and this spatial resolution is unable to capture smaller water bodies that can act as larval habitat.

The presence of the Koga irrigation scheme did not influence malaria cases. The consistent source of water in irrigated areas creates breeding habitat for anopheline mosquitoes, as observed for a comparable large dam project in Ethiopia [94]. The lack of an irrigation effect on malaria in our study may be an example of what Ijumba and Lindsay describe as the “paddies paradox” [95]. There are two possible explanations for this phenomenon in this area. The first factor may be the separation between agricultural land and settlements. The kebeles that are dominated by the Koga irrigation scheme do not have large settlements, as those are located further away in neighboring kebeles that do not have irrigated land cover. Secondly, the Koga irrigation scheme reportedly increased wealth in irrigator households [50]. Increased wealth, better access to health care and education may contribute to a low burden of malaria in this region. Despite high receptivity to malaria because of readily available breeding sites, the spatial separation between irrigation and settlements, in addition to improvements of the socio-economic conditions in the area may have reduced susceptibility and vulnerability of the human population, leading to low malaria cases despite available mosquito habitats.

The utilization of remotely sensed satellite data to study malaria infection patterns comes with limitations. The nature of satellite earth observation data allows us to study the physical properties of the earth surface and measure a variety of factors that influence malaria receptivity. We could not directly measure socio-economic factors like housing, access to health care, and effectiveness of malaria interventions that affect the susceptibility of human populations. In addition, we could not quantify the vulnerability of communities to imported cases. We also could not relate the observed patterns to different species of vector mosquitoes, as entomological data was not available at such a large extent. Particularly in Mecha and Bahir Dar Zuria, the cross-validated R^2^ values from 0.54 to 0.68 indicate that environmental variables alone cannot explain all of the spatial and interannual variation in malaria cases. The importance of non-environmental factors in this study area is also reflected in the strong downward trend of malaria cases due to the impact of recent intervention programs. Because this study focused on assessing different sources of remotely-sensed environmental data, incorporating these socio-economic factors was outside our scope.

We faced additional limitations in classifying settlements via PlanetScope imagery. The spatial resolution of 3 m was sufficient to capture agglomerations of buildings, as well as most of the isolated buildings. However, it was not sufficient to capture some smaller huts with thatched roofs. To capture these buildings, imagery with an even higher spatial resolution would be necessary. Additional research on high resolution settlement mapping could also help to improve downscaled population estimates such as those produced by WorldPop [59] and LandScan [60], which would allow local estimates of malaria incidence in addition to the proportion of outpatients diagnosed with malaria.

## Conclusion

Relationships between spatio-temporal patterns of malaria proportion and environmental variables derived from satellite imagery varied in two landscapes in Ethiopia and were different from results of previous malaria-environment studies conducted at coarser resolution (data summarized at woreda level) and finer (sample households at village level) resolutions. Associations between climate variables and malaria followed the expected pattern with higher temperatures and more rainfall leading to more malaria cases. In both study areas, we found that kebeles with lower vegetation greenness and moisture during the malaria transmission season had the most malaria cases. Malaria was associated with concentrated settlement patterns in one study area, and with low settlement density in the other study area. To some degree, the relationships of these environmental variables with malaria likely reflected indirect relationships with aspects of the social environment such as seasonal migration, water management, and access to health care that affect malaria risk. Recent interventions have lowered malaria incidence and may also have modified some of these malaria-environment associations. These results emphasize that malaria-environment relationships based on remotely sensed environmental indices are contingent on the scale of analysis as well as the geographic setting. Knowledge of the local malaria epidemiology and its connections with physical and human geography is therefore essential for understanding these relationships and applying them to support risk assessment for public health applications.

## Supplementary Information

**Additional file 1**: Supplemental methods describing the SaTScan analyses used for cluster detection.

**Additional file 2**: Supplemental maps of the study areas showing high-resolution land cover and terrain.

**Additional file 3**: Supplemental tables of variable importance for all variables used in each model.

## Data Availability

Satellite data products MODIS, Landsat, and IMGERG data used are publicly available and were accessed via Google Earth Engine (https://code.earthengine.google.com/). PlanetScope data are accessible for researchers via the “Education and Research Program (https://www.planet.com/markets/education-and-research/). Public health data that support the findings of this study are not publicly available, as they were obtained via a data sharing agreement with the Amhara Regional Health Bureau.
