# Supplementary material for: Remote sensing of environmental risk factors for malaria in different geographic contexts
Source: Int J Health Geogr. 2021 Jun 13;20:28. doi: 10.1186/s12942-021-00282-0 (PMC8201719; doi:10.1186/s12942-021-00282-0)
Supplement: Supplementary file 1 — Additional file 1: Supplemental methods describing the SaTScan analyses used for cluster detection. [file 12942_2021_282_MOESM1_ESM.docx]

**Remote sensing of environmental risk factors for malaria in different geographic contexts**

Andrea McMahon^1^, Abere Mihretie^2^, Adem Agmas Ahmed3^,^ Mastewal Lake^4^, Worku Awoke^5^, Michael Charles Wimberly^1^*

1 Department of Geography and Environmental Sustainability, University of Oklahoma, Norman OK, USA

2 Health, Development, and Anti-Malaria Association, Addis Ababa, Ethiopia

3 Malaria Control and Elimination Partnership in Africa, Bahir Dar, Ethiopia

4 Amhara Public Health Institute, Bahir Dar, Ethiopia

5 School of Public Health, Bahir Dar University, Bahir Dar, Ethiopia

* Corresponding author: [mcwimberly@ou.edu](mailto:mcwimberly@ou.edu)

**Additional File 1: Supplementary Methods**

**Satscan Analysis**

To study whether spatial clustering of malaria cases occurs in our study area, we ran spatial scan statistics using SaTScan software. SatScan is designed to detect statistically significant spatial, temporal, or space-time clusters [1]. For our study we used a retrospective, purely spatial Poisson-based scan statistic with an elliptical scan window, which we then ran for each year individually. The SatScan method searches for clusters by exploring multiple elliptical windows of varying location, size, and shapes across the study area.

For each sample ellipse, SaTScan determines the number of observed versus the expected observations, where the number of expected cases is proportional to the population under risk in each area. As no recent population data is available at kebele level, we used annualized outpatient numbers from health facilities within each kebele as indicators for population at risk.

The observed and expected values within each ellipse are then compared to those outside of the ellipse via a likelihood ratio. Scan clusters with significantly higher than expected prevalence, are labeled as high clusters. We refer to these statistically significant clusters of higher than expected malaria cases, as “hotspots”.

The maximum spatial cluster size identifiable was defined as a percentage of the population used in the analysis. We used an upper limit of 50% of the population at risk, as it was recommended by the user guide, and as it is the highest percentage the software will allow [1]. The advantage of choosing a high percentage is that it allows the software to look for clusters of varying size more freely and without cluster size pre-selection bias.

**References**

1. Kulldorff M. SaTScan TM User Guide. 2018.
