# Supplementary material for: Remote sensing of environmental risk factors for malaria in different geographic contexts
Source: Int J Health Geogr. 2021 Jun 13;20:28. doi: 10.1186/s12942-021-00282-0 (PMC8201719; doi:10.1186/s12942-021-00282-0)
Supplement: Supplementary file 2 — Additional file 2: Supplemental maps of the study areas showing high-resolution land cover and terrain. [file 12942_2021_282_MOESM2_ESM.docx]

**Remote sensing of environmental risk factors for malaria in different geographic contexts**

Andrea McMahon^1^, Abere Mihretie^2^, Adem Agmas Ahmed3^,^ Mastewal Lake^4^, Worku Awoke^5^, Michael Charles Wimberly^1^*

1 Department of Geography and Environmental Sustainability, University of Oklahoma, Norman OK, USA

2 Health, Development, and Anti-Malaria Association, Addis Ababa, Ethiopia

3 Malaria Control and Elimination Partnership in Africa, Bahir Dar, Ethiopia

4 Amhara Public Health Institute, Bahir Dar, Ethiopia

5 School of Public Health, Bahir Dar University, Bahir Dar, Ethiopia

* Corresponding author: [mcwimberly@ou.edu](mailto:mcwimberly@ou.edu)

**Additional File 2: Supplementary Maps**


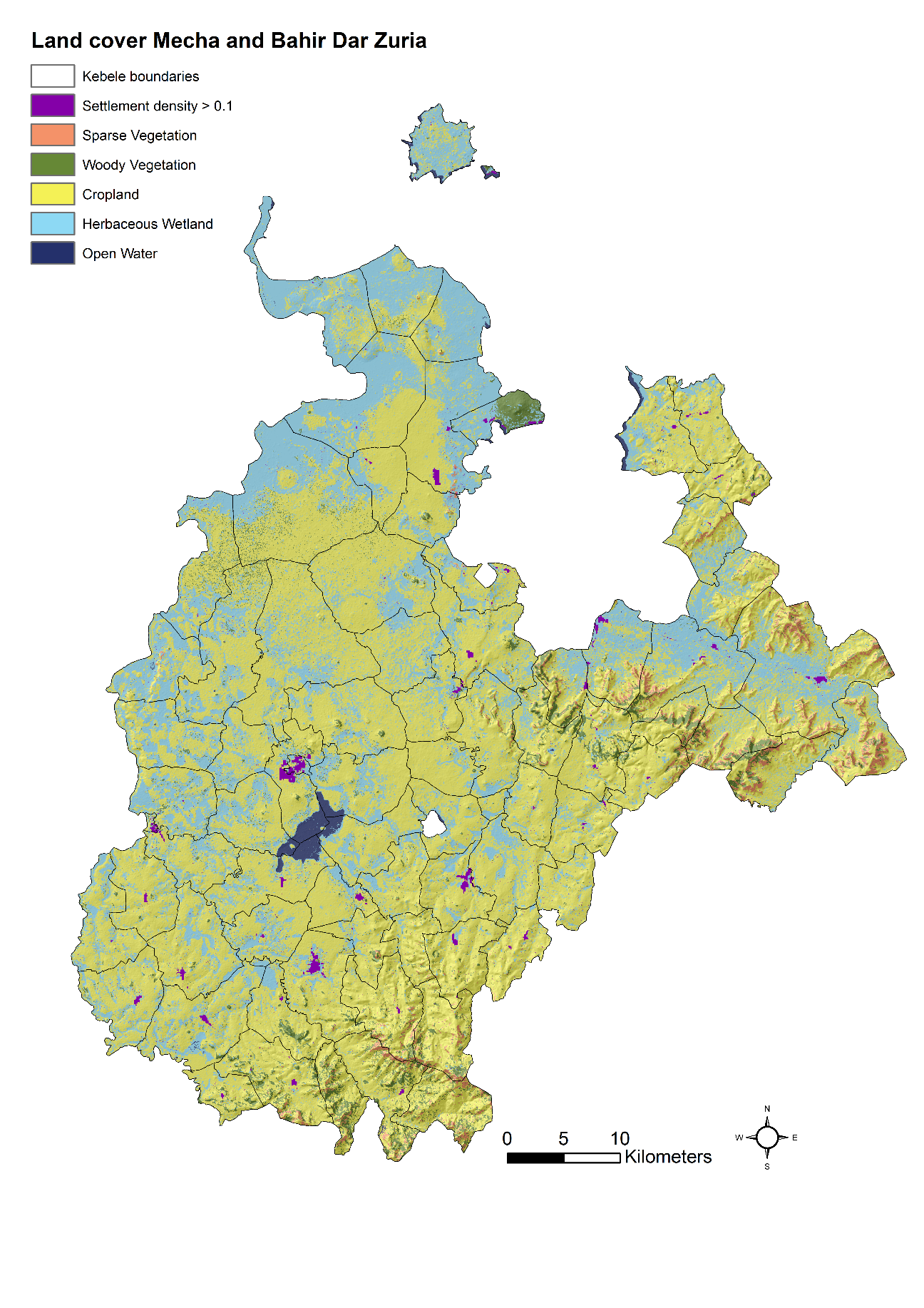


Figure 1: Major land cover classes and hillshade in Mecha and Bahir Dar Zuria. Settlement density is derived based on a classification of buildings via high-resolution PlanetScope imagery. A settlement density value above 0.1 can be considered an agglomeration of buildings. All other land cover classes are taken from Midekisa et al. [1]


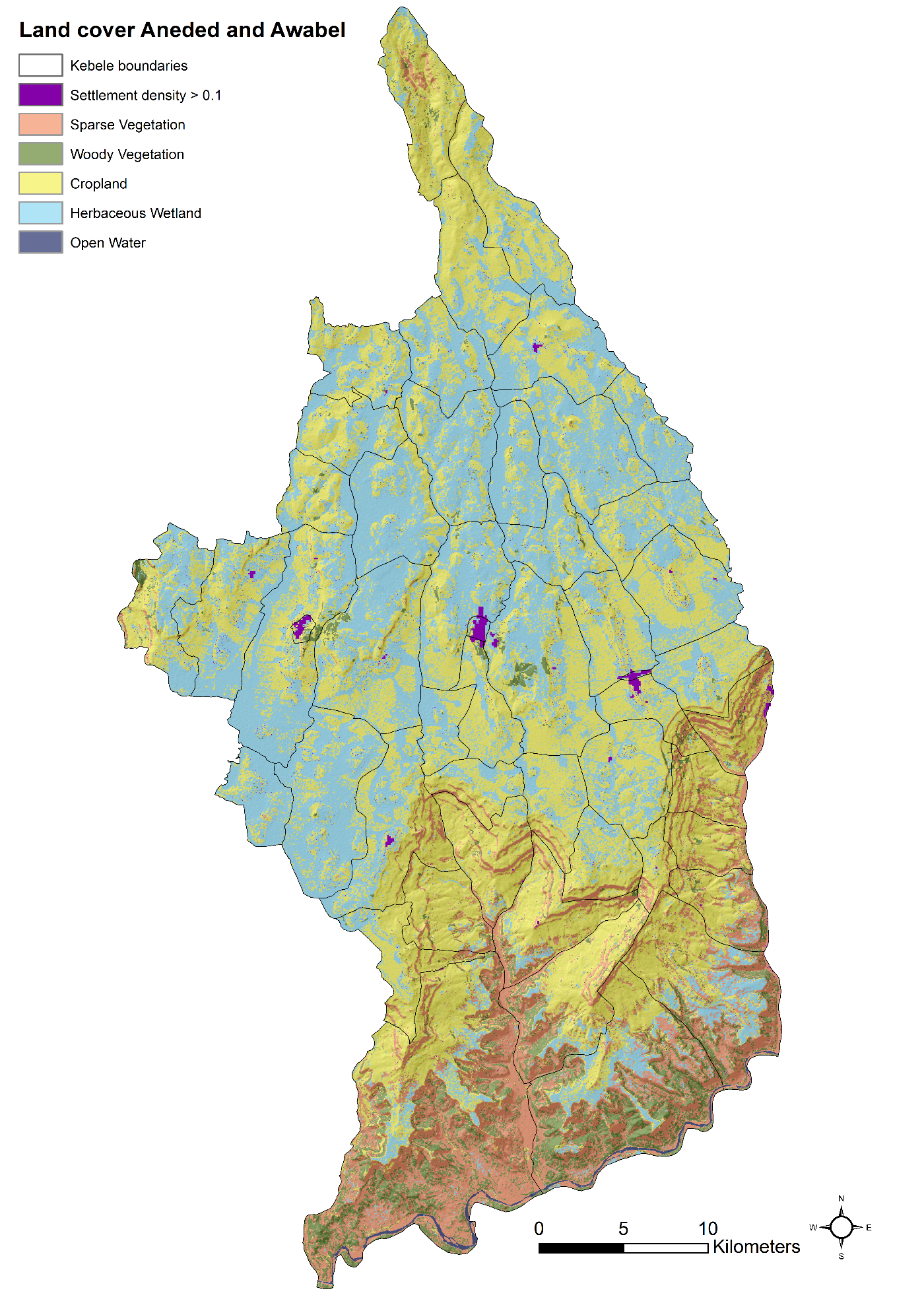


Figure 2:Figure 1: Major land cover classes and hillshade in Aneded and Awabel. Settlement density is derived based on a classification of buildings via high-resolution PlanetScope imagery. A settlement density value above 0.1 can be considered an agglomeration of buildings. All other land cover classes are taken from Midekisa et al. [1]

**References**

1. Midekisa A, Senay GB, Wimberly MC. Multisensor earth observations to characterize wetlands and malaria epidemiology in Ethiopia. Water Resour Res. 2014;50:8791–806.
