# Supplementary material for: Remote sensing of environmental risk factors for malaria in different geographic contexts
Source: Int J Health Geogr. 2021 Jun 13;20:28. doi: 10.1186/s12942-021-00282-0 (PMC8201719; doi:10.1186/s12942-021-00282-0)
Supplement: Supplementary file 3 — Additional file 3: Supplemental tables of variable importance for all variables used in each model. [file 12942_2021_282_MOESM3_ESM.docx]

**Remote sensing of environmental risk factors for malaria in different geographic contexts**

Andrea McMahon^1^, Abere Mihretie^2^, Adem Agmas Ahmed3^,^ Mastewal Lake^4^, Worku Awoke^5^, Michael Charles Wimberly^1^*

1 Department of Geography and Environmental Sustainability, University of Oklahoma, Norman OK, USA

2 Health, Development, and Anti-Malaria Association, Addis Ababa, Ethiopia

3 Malaria Control and Elimination Partnership in Africa, Bahir Dar, Ethiopia

4 Amhara Public Health Institute, Bahir Dar, Ethiopia

5 School of Public Health, Bahir Dar University, Bahir Dar, Ethiopia

* Corresponding author: [mcwimberly@ou.edu](mailto:mcwimberly@ou.edu)

**Additional File 3: Supplementary Results**

Table 1: Mecha and Bahir Dar Zuria variable importance measures. Shown are all variables used to train a Boosted Regression Tree Model, and their variable importance measure (%). Variables are ranked in descending order, according to their contribution in fitting the model.

| Total Malaria | | P. falciparum | | P. vivax | |
| --- | --- | --- | --- | --- | --- |
| Variable | Variable importance % | Variable | Variable importance % | Variable | Variable importance % |
| NDVI trans | 11.07 | NDVI trans | 10.73 | NDVI trans | 17.91 |
| SETMX | 10.64 | PREC rainy | 9.63 | SETMX | 13.25 |
| PREC rainy | 9.30 | NDMI trans | 8.66 | Year | 8.62 |
| Year | 8.97 | Year | 7.35 | NDMI trans | 6.92 |
| NDMI trans | 8.55 | PREC trans | 7.26 | PREC trans | 5.07 |
| PREC trans | 8.34 | SETMX | 7.06 | NDVI dry | 4.90 |
| SPVEG | 5.09 | LST trans | 5.80 | SETME | 4.87 |
| WETL | 4.91 | WETL | 4.74 | NDMI rainy | 4.75 |
| LST trans | 4.39 | SPVEG | 4.62 | NDMI dry | 4.65 |
| NDMI rainy | 3.53 | NDMI rainy | 4.12 | NDVI rainy | 4.08 |
| HAND | 3.17 | NDVI rainy | 3.57 | PREC rainy | 3.86 |
| NDVI dry | 3.09 | PREC dry | 3.52 | CROP | 3.26 |
| NDVI rainy | 2.48 | WOODY | 3.40 | SPVEG | 3.16 |
| SETME | 2.48 | NDVI dry | 3.14 | LST trans | 3.01 |
| PREC dry | 2.45 | DISTSW | 3.09 | HAND | 2.60 |
| WOODY | 2.41 | NDMI dry | 2.41 | PREC dry | 1.92 |
| NDMI dry | 2.33 | LST dry | 2.26 | WETL | 1.51 |
| LST dry | 1.55 | HAND | 2.24 | LST dry | 1.41 |
| WATER | 1.52 | LST rainy | 2.02 | WATER | 1.22 |
| LST rainy | 1.50 | SETME | 1.76 | WOODY | 1.15 |
| DISTSW | 1.25 | CROP | 1.72 | DISTSW | 1.02 |
| CROP | 0.94 | WATER | 0.88 | LST rainy | 0.84 |
| IRRI | 0.04 | IRRI | 0.01 | IRRI | 0.04 |

Table 2: Aneded and Awabel variable importance measures. Shown are all variables used to train a Boosted Regression Tree Model, and their variable importance measure (%). Variables are ranked in descending order, according to their contribution in fitting the model.

| Total malaria | | P. falciparum | | *P. vivax* | |
| --- | --- | --- | --- | --- | --- |
| Variable | Variable importance % | Variable | Variable importance % | Variable | Variable importance % |
| NDMI dry | 43.10 | NDMI dry | 41.19 | NDMI dry | 36.70 |
| WOODY | 11.03 | LST trans | 14.56 | NDVI trans | 6.69 |
| LST trans | 10.52 | WOODY | 11.10 | WOODY | 6.44 |
| NDVI trans | 6.58 | NDVI trans | 7.09 | LST trans | 6.11 |
| SETME | 5.56 | SETME | 6.74 | PREC trans | 5.84 |
| SPVEG | 4.73 | CROP | 3.06 | SETMX | 5.55 |
| CROP | 2.63 | LST rainy | 2.99 | PREC rainy | 4.34 |
| PREC trans | 2.48 | SPVEG | 2.71 | SETME | 3.99 |
| LST rainy | 2.20 | PREC trans | 1.73 | SPVEG | 2.74 |
| NDMI trans | 1.81 | LST dry | 1.65 | NDVI dry | 2.67 |
| LST dry | 1.30 | NDMI trans | 1.61 | PREC dry | 2.32 |
| PREC rainy | 1.23 | Year | 1.12 | NDMI trans | 2.32 |
| NDVI dry | 1.13 | NDVI rainy | 0.72 | CROP | 2.19 |
| SETMX | 0.85 | WATER | 0.65 | HAND | 2.09 |
| NDMI rainy | 0.82 | NDMI rainy | 0.57 | DISTSW | 2.02 |
| PREC dry | 0.72 | DISTSW | 0.56 | LST rainy | 1.93 |
| Year | 0.70 | PREC rainy | 0.45 | NDMI rainy | 1.84 |
| DISTSW | 0.61 | WETL | 0.38 | LST dry | 1.75 |
| WATER | 0.60 | PREC dry | 0.38 | NDVI rainy | 1.10 |
| NDVI rainy | 0.55 | NDVI dry | 0.38 | WETL | 1.04 |
| HAND | 0.46 | HAND | 0.19 | Year | 0.32 |
| WETL | 0.41 | SETMX | 0.17 | WATER | 0.00 |
